# Identification of biomarkers complementary to homologous recombination deficiency for improving the clinical outcome of ovarian serous cystadenocarcinoma

**DOI:** 10.1002/ctm2.399

**Published:** 2021-05-18

**Authors:** Zhiwen Shi, Qingguo Zhao, Bin Lv, Xinyu Qu, Xiao Han, Hongyan Wang, Junjun Qiu, Keqin Hua

**Affiliations:** ^1^ Obstetrics and Gynecology Hospital Fudan University Shanghai China; ^2^ State Key Laboratory of Genetic Engineering, MOE Key Laboratory of Contemporary Anthropology, and Collaborative Innovation Center for Genetics & Development, School of Life Sciences Fudan University Shanghai China; ^3^ Shanghai Key Laboratory of Female Reproductive Endocrine‐Related Diseases Shanghai China

**Keywords:** cGAS‐STING, *CXCL11*, HRD, ovarian cancer, PARPi, TIME

## Abstract

Ovarian cancer patients with homologous recombination deficiency (HRD) tumors would benefit from PARP inhibitor (PARPi) therapy. However, patients with HRD tumors account for less than 50% of the whole cohort, so new biomarkers still need to be developed. Based on the data from the SNP array and somatic mutation profiles in the ovarian cancer genome, we found that high frequency of actionable mutations existed in patients with non‐HRD tumors. Through transcriptome analysis, we identified that a downstream target of the cGAS‐STING pathway, *CXCL11*, was upregulated in HRD tumors and could be used as a predictor of survival outcome. Further comprehensive analysis of the tumor immune microenvironment (TIME) revealed that *CXCL11* expression signature was closely correlated with cytotoxic cells, neoantigen load and immune checkpoint blockade (ICB). Clinical trial data confirmed that the expression of *CXCL11* could be used as a biomarker for anti‐PD‐1/PD‐L1 therapy. Finally, in vivo and in vitro experiments showed that cancer cells with PARPi treatment increased the expression of *CXCL11*. Collectively, our study not only provides biomarkers of ovarian cancer complementary to the HRD score but also introduces a potential new perspective for identifying prognostic biomarkers of immunotherapy.

## Graphical abstract

• There is a high proportion of actionable gene mutations in HR proficient patients with OSC.

• *CXCL11* can be used as a biomarker for immunotherapy.

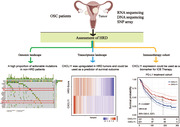

**Abbreviations**
CNV: copy number variation
CXCL11: C‐X‐C motif chemokine 11
DEGs: differentially expressed genes
GEO: Gene Expression Omnibus
GSEA: gene set enrichment analysis
HR: homologous recombination repair
HRD: homologous recombination deficiency
ICB: immune checkpoint blockade
LOH: loss of heterozygosity
LST: largescale state transitions
MSI: microsatellite instability
OSC: ovarian serous cystadenocarcinoma
PARPi: PARP inhibitor
TAI: telomeric allelic imbalance
TCGA: the Cancer Genome Atlas
TIME: tumor immune microenvironment
TPM: transcripts per million

## 1 INTRODUCTION

The incidence rate of ovarian cancer ranks third among female genital tract malignancies, but its mortality rate ranks first.^1^ About 70% of ovarian cancer patients have advanced cancer at the time of initial diagnosis, as there are no obvious symptoms in the initial stage of ovarian cancer.^2^ Ovarian serous cystadenocarcinoma (OSC), a common type of ovarian cancer, accounts for about 90% of all ovarian cancers^3^ and it is prone to peritoneal metastasis early and chemotherapy resistance. According to statistics, the 5‐year survival rate of ovarian cancer patients is only 30–45%.^4^ The major reason for the poor prognosis of ovarian cancer is lack of effective means of early diagnosis and prognostic indicators. Discovering specific biomarkers for early screening of ovarian cancer and new therapeutic targets for ovarian cancer are the current focus of ovarian cancer research.

Emerging clinical trials have revealed the clinical value of homologous recombination deficiency (HRD) in ovarian cancer. Homologous recombination repair (HR) plays an important role in DNA repair mechanisms. *BRCA* (*BRCA1/2*), *RAD51* (*RAD51B/C/D*), *BRIT1*, etc. are key components of HR‐mediated DNA repair.^5^, ^6^, ^7^ HRD tumors were recorded for the first time in patients that harbored germline mutations of *BRCA* gene. In the phase 3 PAOLA‐1 (PAOLA‐1/ENGOT‐ov25) trial, the addition of maintenance olaparib provided a significant progression‐free survival benefit, which was substantial in patients with HRD tumors, including those without a *BRCA* mutation.^8^ The molecular mechanism of HRD is not fully understood. Current studies have found that mutations in genes, including *BRCA* gene mutations, involved in the HR signaling pathway can only explain about 14.1% of HRD ovarian cancer patients.^9^ Therefore, research on transcriptome characteristics of HRD patients may fill this gap. Although several studies have investigated the relationship between the transcriptome and tumor genome instability,^10^, ^11^ HRD‐associated RNAs and their clinical significance in ovarian cancer still remain largely unexplored. Moreover, HRD is present in less than 50% of serous ovarian tumors,^12^ so new biomarkers need to be developed for molecular typing of ovarian cancer patients with non‐HRD tumors.

For the first time in the present study, by taking advantage of both the Cancer Genome Atlas (TCGA)/Gene Expression Omnibus (GEO) database and the algorithm for quantifying HRD scores, we found that high frequency of actionable mutations existed in patients with non‐HRD tumors. Through transcriptome analysis, we identified and validated the C–X–C motif chemokine 11 (*CXCL11*) that predicted the survival and prognosis of OSC patients. Furthermore, we discovered a relationship between *CXCL11* expression and tumor immune microenvironment (TIM`E), including cytotoxic cells, neoantigen load, and immune checkpoint blockade (ICB). Moreover, high *CXCL11* expression was able to be used as a biomarker for anti‐PD‐1/PD‐L1 therapy, and the predictive effect of *CXCL11* was better than that of PD‐1/PD‐L1. Finally, in vivo and in vitro experiments confirmed that olaparib could upregulate the expression of *CXCL11* in ovarian cancer cell lines. Our research perspectives and methods provide a possible direction for molecular typing of ovarian cancer. The results of this study may be valuable for understanding the relationship between HRD and TIME and improving the clinical outcome of patients receiving anti‐PD‐1/PD‐L1 therapy.

## 2 MATERIALS AND METHODS

### 2.1 Data collection and processing

OSC patients’ RNA sequencing data, somatic mutation data, SNP array data, and corresponding clinical follow‐up information were downloaded from the publicly available TCGA database (https://portal.gdc.cancer.gov) and the NCBI GEO database.^13^ RNA sequencing data were normalized as transcripts per million (TPM) by using the R. SNP array data were processed using Affymetrix Power Tools and PennCNV. The somatic mutation counts, copy number variation (CNV), fraction genome altered scores (percentage of copy number altered chromosome regions out of measured regions), and microsatellite instability (MSI) sensor score were obtained from the cBioPortal database (https://www.cbioportal.org/). In total, 348 TCGA samples data were extracted; 296 GEO samples data were extracted (GSE140082 and GSE30161). The transcriptome profile and clinical information from immunotherapy cohorts were obtained from Imvigor210.^14^, ^15^

### 2.2 HRD score analysis

Loss of heterozygosity (LOH) was defined as the number of counts of chromosomal LOH regions shorter than whole chromosome and longer than 15 Mb.^16^ Largescale state transitions (LST) were defined as chromosome breakpoint (change in copy number or allelic content) between adjacent regions each of at least 10 Mb obtained after smoothing and filtering shorter than 3 Mb small‐scale CNV.^17^ Telomeric allelic imbalance (TAI) was defined as the number of regions with allelic imbalance that extend to the subtelomere but do not cross the centromere.^18^ The HRD score was defined as the sum of TAI, LST, and LOH scores.^19^ The HRD score of each patient is shown in Table S1.

### 2.3 Genomic landscape and neoantigen load

The datasets of the somatic mutations for OSC in TCGA were obtained from the MC3 TCGA dataset and analyzed using the TCGA mutations package of R.^20^ Somatic mutation alterations were analyzed by using the maftools package of R.^21^ The four‐digit HLA type for each sample was inferred using POLYSOLVER (POLYmorphic loci reSOLVER), which uses a normal tissue .bam file as input and employs a Bayesian classifier to determine genotype.^22^ By comparing to matched tumor bams, POLYSOLVER also identified HLA mutations. Neo‐epitopes were predicted for each patient by defining all novel amino acid 9mers and 10mers resulting from mutation in expressed genes (median >10 TPM in the tumor type) and determining whether the predicted binding affinity to the patient's germline HLA alleles was <500 nM using NetMHCpan.^23^, ^24^, ^25^, ^26^ The Neoantigen load of each patient is shown in Table S2.

### 2.4 Gene set enrichment analysis (GSEA)

RNA‐seq data (raw counts) analysis was conducted using the “edgeR” package of R.^27^ Fold change >1.5, adj. *p* < .05, TPM >1, and genes with the first 75% of median absolute deviation were set as the cutoffs to screen for differentially expressed genes (DEGs). Heatmaps and clustering were generated using an open‐source web tool ClustVis.^28^ GSEA was performed using GSEA software from the Broad Institute (MIT, Cambridge, MA) to identify differential signaling pathways in different groups.^29^ The normalized enrichment score was calculated for each gene set. GSEA results with a nominal *p* < .05 were considered significant.

### 2.5 Identification of prognostic related genes associated with HRD score

**FIGURE 1:**
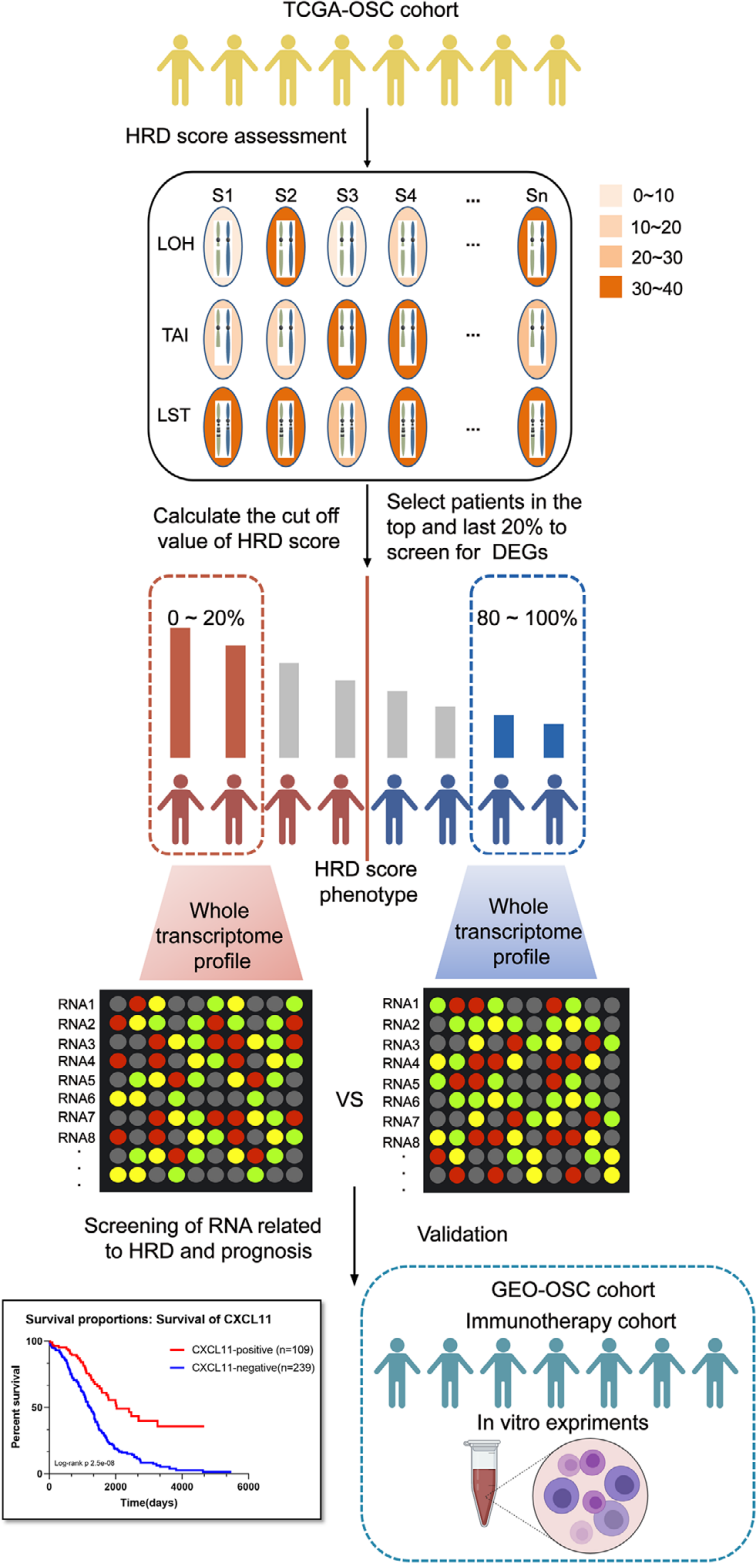
Computational overview of homologous recombination deficiency (HRD)‐related RNAs detection. Columns reflect ovarian cancer samples, and the rows reflect three biomarkers of the HRD score. Color reflects the scores for each biomarker in each sample. HRD‐related RNAs were detected by comparing the RNA expression profile between the top 20% patients with high HRD scores and the bottom 20% patients with low HRD scores

Univariable Cox regression analysis was performed to select the prognostic related genes using the computing environment R with the survival package.^30^ The prognosis‐related genes with a *p*‐value < .05 in the univariate Cox regression analysis were considered as candidate variables. The results were further analyzed through the LASSO regression approach to seek a balance between the maximization of prediction accuracy and the minimization of interpretation.^31^ The screening process is shown in Figure 1.

### 2.6 Immune cells infiltration in bulk tumor gene expression data

In order to study the enrichment of immune cells in *CXCL11‐negative* and *CXCL11‐positive* groups, we used TIMER,^32^ an efficient algorithm for predicting immune cell infiltration of bulk tumor gene expression data (https://cistrome.shinyapps.io/timer/). For each sample, TIMER quantified the relative abundance of six types of infiltrating immune cells, including T cells, B cells, macrophages, neutrophiles, and dendritic cells.

### 2.7 Cells and culture

A2780 and A2780cisR (cisplatin resistant) human ovarian cell lines were gifts from Fudan University Shanghai Cancer Center. IOSE‐80 and HEY‐T30 ovarian cell lines originated from a gift from Dr Luopei Guo (Obstetrics and Gynecology Hospital of Fudan University). ES‐2, SKOV3, OVCAR3, and CAOV3 ovarian cell lines were purchased from GeneChem (Shanghai, China). All cell lines were cultured according to ATCC guidelines at 37°C in a 5% CO2 incubator. The olaparib (Selleck, catalog number S1060) was dissolved in DMSO, and the final concentration of DMSO in the medium was 0.1%. After the cells were plated for 24 h, cells were overlaid with (0, 2, 10, 25, 50 μM) olaparib‐conditioned medium and harvested for 24 h.

### 2.8 Real time‐quantitative PCR (RT‐qPCR) analysis

For cDNA synthesis, 1 μg total RNA was processed using the HiScript RT SuperMix for qPCR (+gDNA wiper) kit (Vazyme). The ChamQ Universal SYBR qPCR Master Mix (Vazyme) was used for the thermocycling reaction. The RT‐qPCR analysis was carried out in triplicate times. Primer sequences were as follows:

*Beta‐ACTIN*: Forward: 5′‐GTGGCCGAGGACTTTGATTG‐3′,
Reverse: 5′‐CCTGTAACAACGCATCTCATATT‐3′,
*CXCL11*: Forward: 5′‐GACGCTGTCTTTGCATAGGC‐3′,
Reverse: 5′‐GGATTTAGGCATCGTTGTCCTTT‐3′.

## 3 RESULTS

### 3.1 HRD score significantly correlated with the prognosis and molecular characteristics of TCGA‐OSC cohort

**FIGURE 2:**
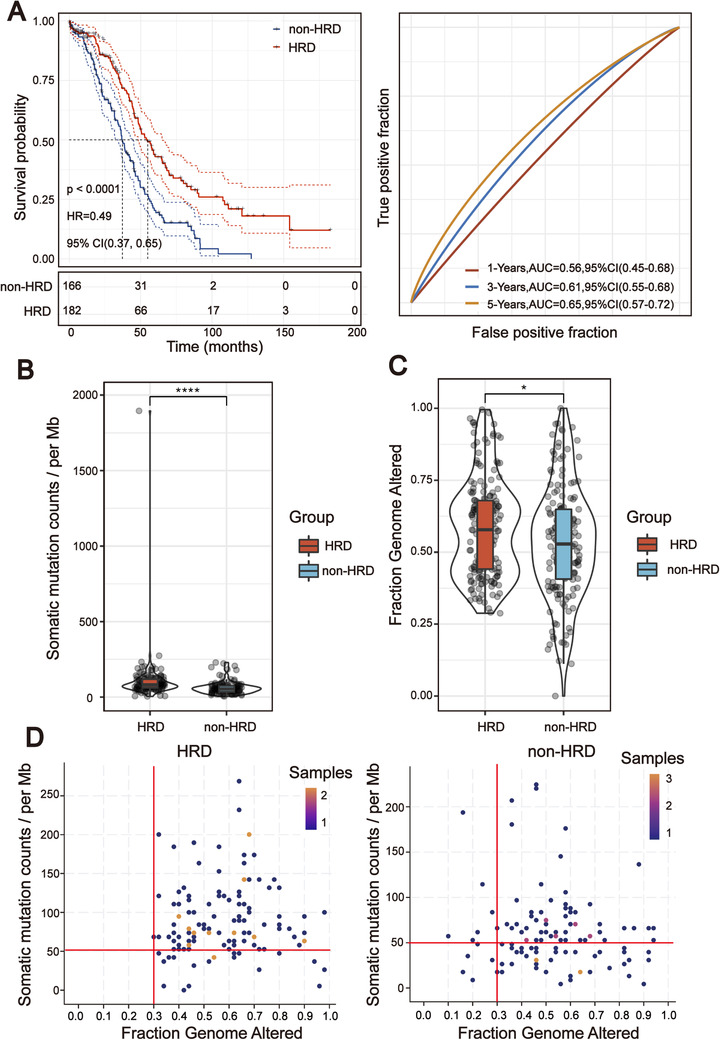
Homologous recombination deficiency (HRD) score was significantly correlated with the prognosis and molecular characteristics of TCGA‐OSC cohort. (A) Kaplan–Meier estimates of overall survival of patients with the HRD or non‐HRD tumors calculated by the HRD score in the TCGA‐OSC cohort. On the right are the AUC curves of HRD score in TCGA‐OSC cohort. (B) Violin plot of somatic mutations in the HRD and non‐HRD groups. Somatic mutation counts in the HRD group were significantly higher than those in the non‐HRD group (Wilcoxon signed rank test, ****p < .0001). (C) Violin plot of fraction genome altered in the HRD and non‐HRD groups (Wilcoxon signed rank test, *p < .05). (D) Two‐dimensional plan of fraction of the genome and somatic mutation counts in different subgroups (Kolmogorov–Simonov test, *p* < .01)

According to the HRD algorithm, LOH, TAI, and LST were used as the basis for calculating the HRD score. Optimal cutoff scores were determined by assessing the score that had the minimum *p*‐value of the log‐rank test (Figure 2A). HRD status was defined as HRD if HRD score was >57; HRD status was defined as non‐HRD if HRD score was ≤57. The Kaplan–Meier survival curve (Figure 2A) showed that overall survival (OS) of patients in the HRD group is much longer than the cases in the non‐HRD group; hazard ratio (HR) = 0.49 (0.37, 0.65), log‐rank test, *p* < .00001. Subsequently, we investigated the correlation between the HRD score and other hallmarks of genomic instability, including somatic mutation counts, fraction genome altered, and MSI. The median value of somatic cumulative mutations in the HRD group was significantly higher than that in the non‐HRD group (Wilcoxon signed rank test, *p* < .0001; Figure 2B). We next compared the fraction genome altered scores between the HRD and non‐HRD groups. As shown in Figure 2C, the fraction genome altered scores in the HRD group were higher than those in the non‐HRD group (Wilcoxon signed rank test, *p* < .05; Figure 2C). The plot of “somatic mutation counts versus fraction genome altered” clearly showed that the distribution of points in the HRD group was concentrated in the upper right of the coordinate system, while that of the non‐HRD group was scattered (Figure 2D). The results of transcriptome level analysis were consistent with those at the genome level: through GSEA analysis, it was found that the three signal pathways with the most significant differences between the two groups were DNA replication, homologous recombination, and mismatch repair (Figure S1A). There was no difference in the MSI status of the two groups (Figure S1B).

### 3.2 Genomic landscape of non‐HRD group showed a high proportion of actionable mutations in *NF1* and *CDK12*

**FIGURE 3:**
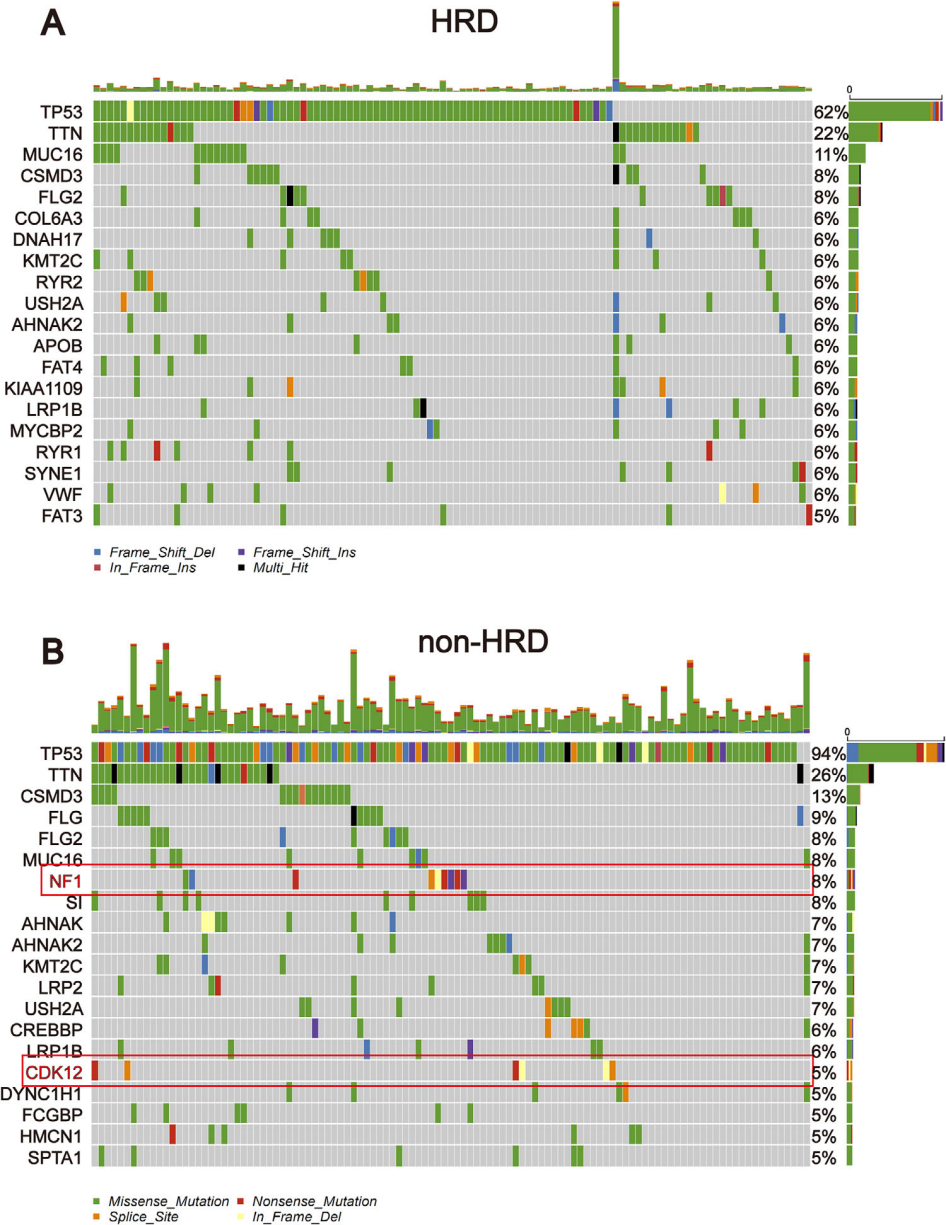
Mutational landscape of TCGA‐OSC cohort stratified by the homologous recombination deficiency (HRD) and non‐HRD groups. (A) Genetic profile of the HRD OSC patients. (B) Genetic profile of the non‐HRD OSC patients. The genes in the red box are actionable genes

Genomic characteristics, such as the oncogene activation (e.g., *ERBB2* amplification, *EGFR* tyrosine kinase mutation) and inactivation of tumor suppressor genes (e.g., MMR, *BRCA1/2*) have shown a strong correlation with clinical response to target therapy. Therefore, we compared the genomic mutational landscape between the HRD and non‐HRD groups. The results showed that the genomic landscape of non‐HRD group was significantly different from that of the HRD group. Only nine of the top 20 genes with the highest mutation rate in the two groups overlapped (Figure S1C). The mutational landscapes of these two subgroups displayed a distinct mutation ratio in *TP53* (94.0% [non‐HRD] vs. 62% [HRD]), and the mutation classification of the non‐HRD group was more abundant, including a higher proportion of frameshift del, nonsense mutation, and so on (Figure 3A,B). Through the screening of actionable genes in the OncoKB database (https://www.oncokb.org/actionableGenes), among the 20 genes with the highest mutation frequency in the non‐HRD group, two genes were biomarkers for targeted drugs (*NF1* and *CDK12*). Moreover, most of the variant classifications of these two genes were those affecting gene structure. Patients with mutations in these two genes accounted for 13% of the non‐HRD group. The mutational landscapes of HR genes in the two subgroups also exhibited a distinct difference. HR gene mutations in the HRD group were mainly concentrated in *BRCA1/2* (7%), while the non‐HRD group was scattered across different HR genes (Figure S2A,B). We also compared the CNV of HR genes in the two subgroups, and observed *BRCA2* homozygous deletion was only present in the HRD group (Figure S2C).

### 3.3 *CXCL11* expression associated with the HRD score and its prognostic value in OSC

**FIGURE 4:**
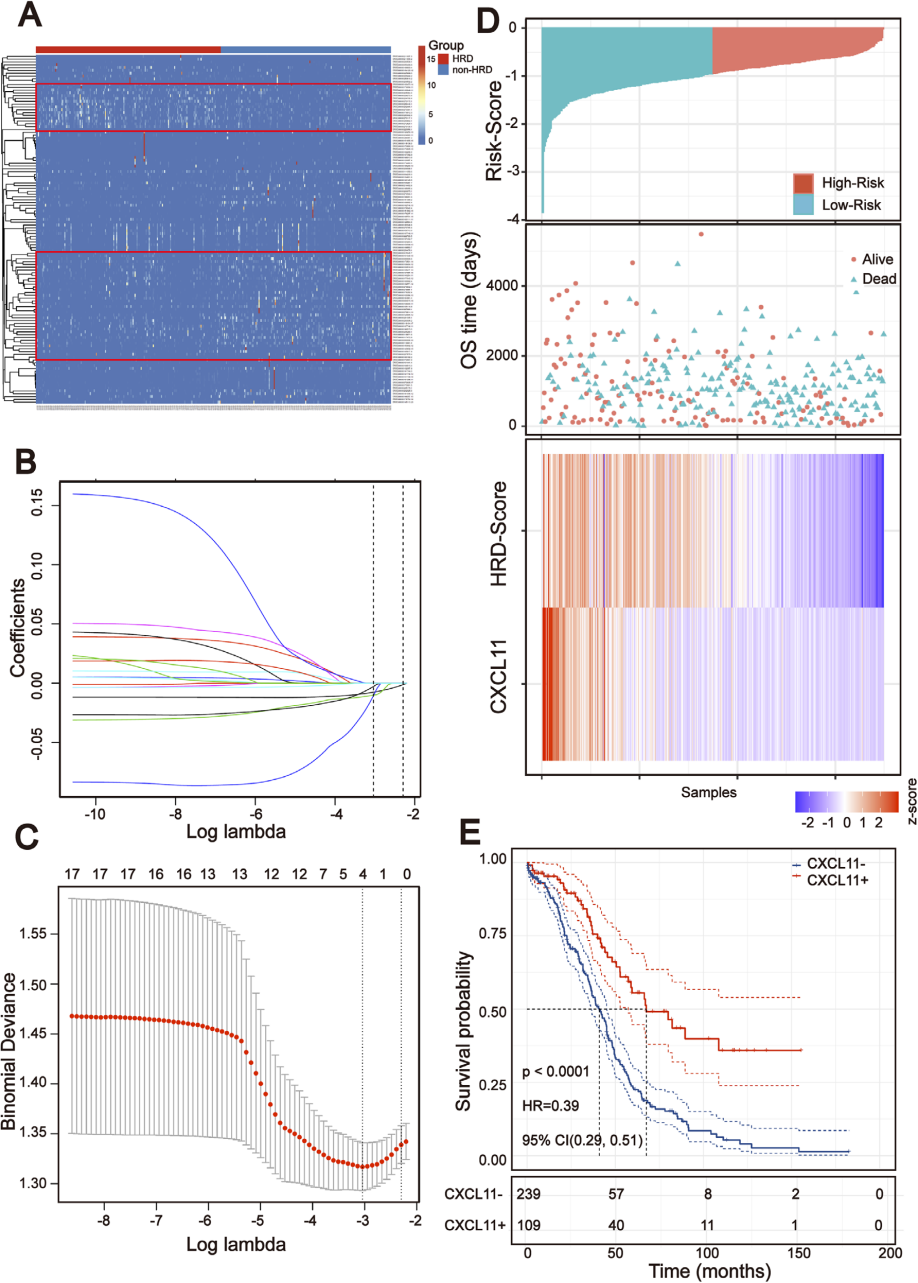
Screening prognosis related RNA based on the homologous recombination deficiency (HRD) score. (A) Unsupervised clustering of 348 OSC patients based on the expression pattern of 124 differentially expressed genes (DEGs). (B and C) Lasso coefficient profiles of the 17 prognosis‐associated HRD genes. (D) Heatmap of the signature consisting of the HRD score and the *CXCL11* expression signature based on the Cox coefficients. Patients were divided into high‐risk and low‐risk groups and the median risk score was utilized as the cutoff value. (E) Kaplan–Meier estimates of overall survival of patients with the *CXCL11*‐positive or *CXCL11*‐negative tumors in the TCGA‐OSC cohort (log‐rank test)

To identify RNAs associated with the HRD score, the TCGA‐OSC cohort was sorted in ascending order of HRD scores, and the last 20% (*n* = 70) and the top 20% (*n* = 70) of the patients were chosen to identify DEGs. Utilizing the egdeR method, a total of 124 DEGs were screened out. Among them, 38 RNAs were found to be upregulated and 86 to be downregulated in the HRD group. Then, 124 differentially expressed RNAs were used to perform unsupervised cluster analysis on 348 TCGA‐OSC samples. As shown in Figure 4A, we found that not all DEGs clustered the HRD and non‐HRD groups well in the entire TCGA‐OSC cohort. Only the DEGs in the red block region were able to cluster the HRD and non‐HRD groups well. To further screen out DEGs related to the HRD score and prognosis of the patients, the univariate analysis was conducted in the 124 DEGs for the whole TCGA‐OSC cohort. A total of 17 genes with prognostic potentiality were identified by the univariate analysis and log‐rank test (*p* < .05). The 17 HRD‐related genes were then subjected to Lasso–Cox proportional hazards regression and 10‐fold cross‐validation to identify the best gene model. The Lasso coefficient profile plot was produced against the log (lambda) sequence, and the minimize *k* method resulted in one optimal coefficient (Figure 4B,C). C–X–C motif chemokine ligand 11 (*CXCL11*), a downstream target of the cGAS‐STING pathway, reached the optimal regression efficiency to speculate the prognostic ability. A heatmap of *CXCL11* expression and the HRD score and the scatterplot of OS with corresponding risk scores are illustrated in Figure 4D. Kaplan–Meier analysis displayed that the survival outcomes of TCGA‐OSC patients with high *CXCL11* expression (*CXCL11*‐positive) were significantly better than patients with low *CXCL11* expression (*CXCL11*‐negative) (HR = 0.39 [0.29, 0.51], log‐rank test *p* < .00001) (Figure 4E). To verify whether the *CXCL11* expression signature has similar prognostic value in different OSC cohorts, we further confirmed this phenomenon in two independent OSC cohorts in the GEO database (including GSE140082 and GSE30161): results from Kaplan–Meier analysis also showed that patients in the *CXCL11*‐positive group demonstrated a better prognosis than those in the *CXCL11*‐negative group (Figure S3A,B).

### 3.4 Comparison of immune cells infiltration within *CXCL11*‐positive and *CXCL11*‐negative groups

**FIGURE 5:**
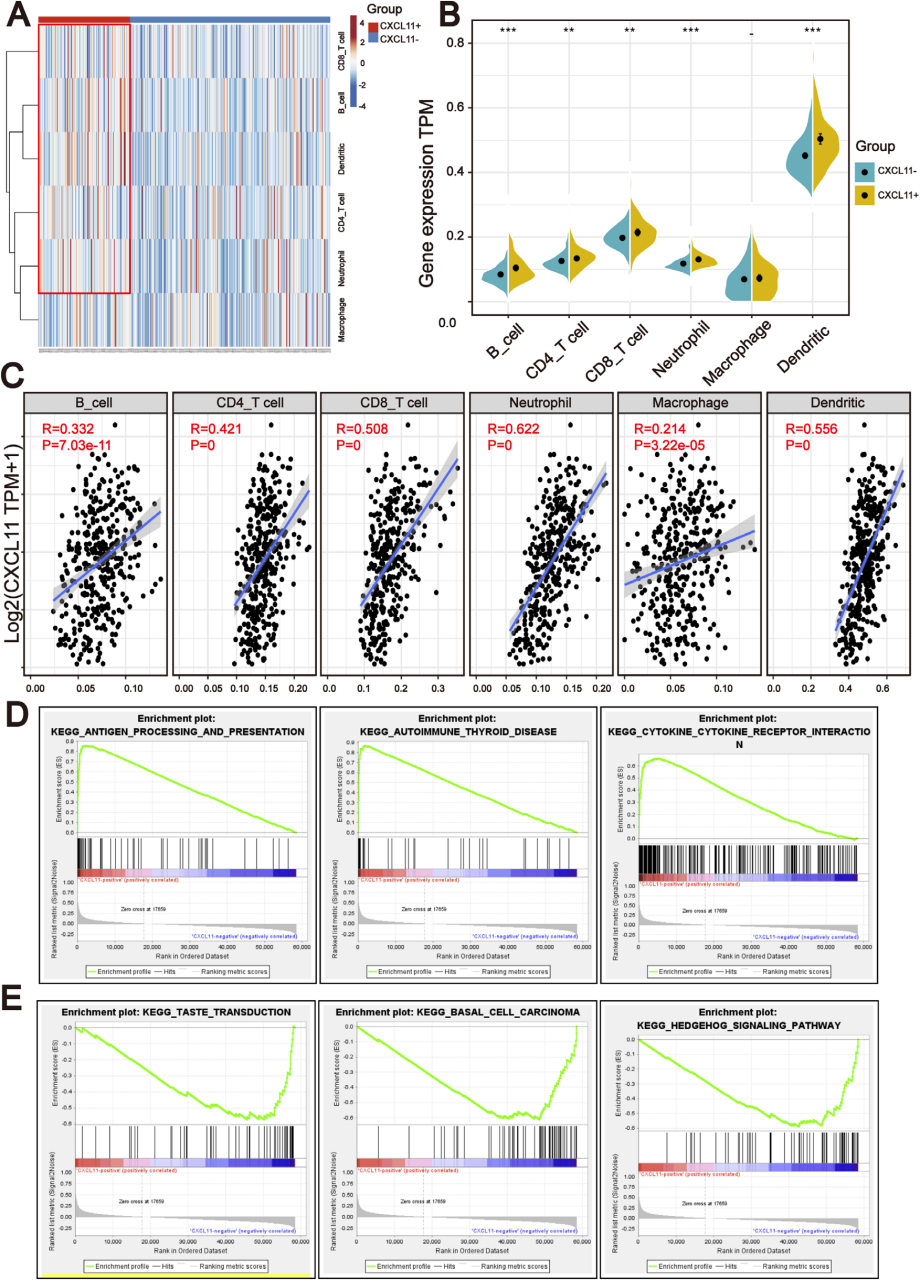
*CXCL11* expression signature was associated with the immune infiltration. (A) TIMER analysis identified the relative infiltration of six types of immune cell subpopulations with different *CXCL11* subgroups. (B) Violin plot of immune cell subpopulations in the *CXCL11*‐positive and *CXCL11*‐negative groups (Wilcoxon signed rank test, **p < .01, ***p < .001). (C) Correlation between the *CXCL11* expression signature and immune cell subpopulations in the TCGA‐OSC cohort. (D) GSEA identified that antigen processing and presentation, autoimmune thyroid and cytokine receptor interaction signaling pathways were upregulated in the *CXCL11*‐positive group compared to the *CXCL11*‐negative group. (E) GSEA identified that taste transduction, basal cell carcinoma, and hedgehog signaling pathways were upregulated in the *CXCL11*‐negative group compared to the *CXCL11*‐positive group

The expression of cytokines/chemokines is essential for attracting immune cells,^33^ suggesting that tumor infiltrating immune cells might be different in the *CXCL11*‐positive and *CXCL11*‐negative groups. To validate this assumption, the TIMER algorithm^34^ was applied to estimate enrichment of various immune cell types within different subgroups. We developed a heatmap with TIMER results to visualize the relative abundance of six immune infiltrating cell subpopulations from the TCGA‐OSC cohort (Figure 5A). As depicted in the heatmap, there were significant differences in immune cell infiltration between the two subgroups. Antitumor lymphocyte cell subpopulations, such as CD4^+^/CD8^+^ T cells and dendritic cells were enriched in the *CXCL11*‐positive group (Wilcoxon signed rank test, *p* < .01). The neutrophils were also enriched in the *CXCL11*‐positive group (Wilcoxon signed rank test, *p* < .001) (Figure 5B). We then investigated the correlation of immune cell infiltration with the expression of *CXCL11* by spearman correlation coefficients. The results revealed that the expression of *CXCL11* was significantly associated with immune cell infiltration in the TCGA‐OSC cohort (Figure 5C). We also further analyzed the correlation between the immune cell infiltration signal and the expression of *CXCL11* in the TCGA pan‐cancer cohorts and found similar results (Figure S3C).

Furthermore, GSEA on the gene expression profile of the *CXCL11*‐positive group against the *CXCL11*‐negative group revealed the *CXCL11* expression signature‐related biological signaling pathway. Genes involved in antigen processing and presentation, autoimmune thyroid and cytokine receptor interaction signaling pathways were the most significantly enriched in the *CXCL11*‐positive group (Figure 5D). However, taste transduction, basal cell carcinoma, and hedgehog signaling pathways were enriched in the *CXCL11*‐negative group (Figure 5E).

### 3.5 *CXCL11* expression associated with molecules in antigen processing and presentation pathway

**FIGURE 6:**
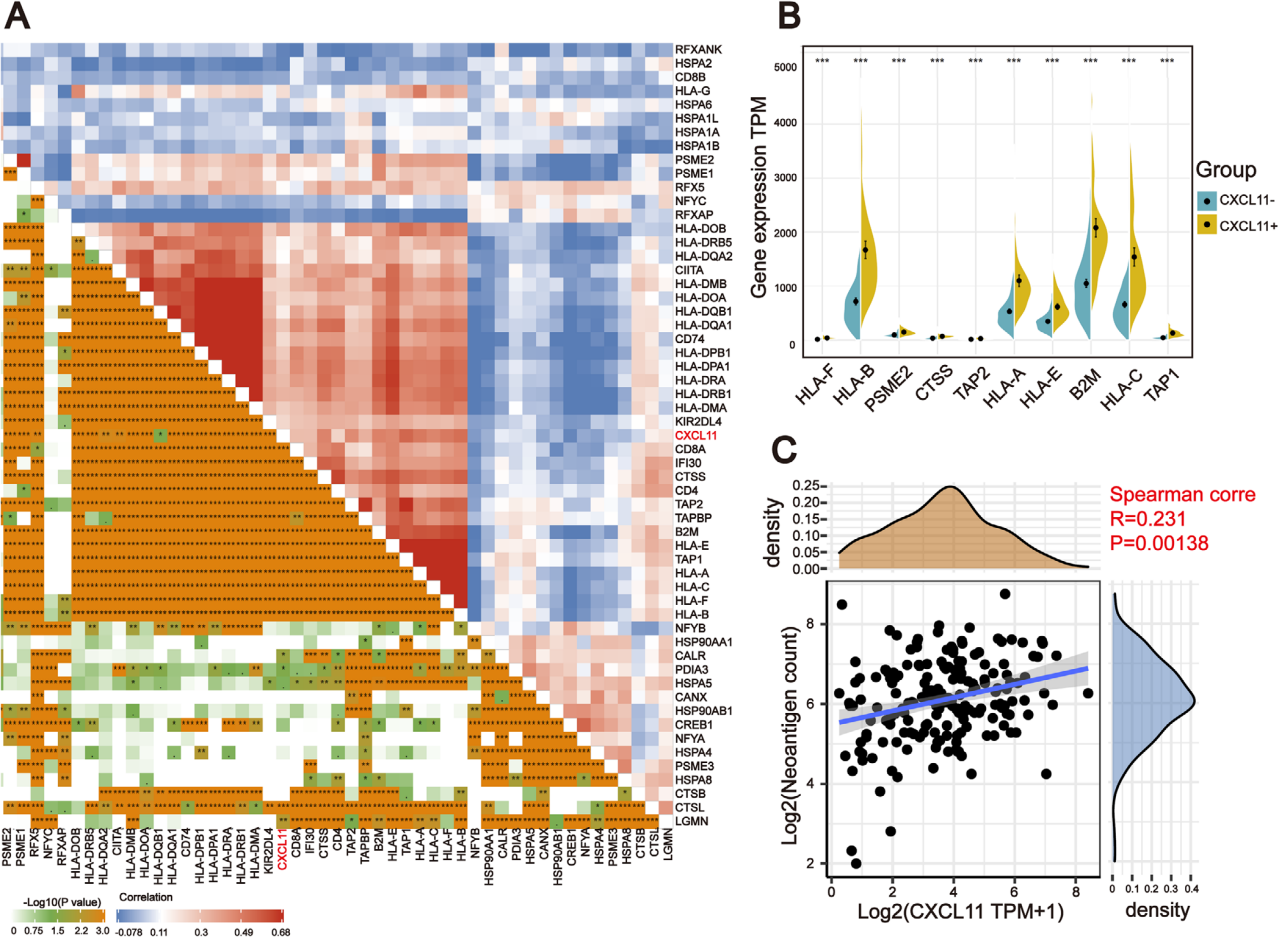
Correlation between the expression of *CXCL11* and antigen‐related genes. (A) Correlation between the *CXCL11* expression signature and antigen‐related genes in the TCGA‐OSC cohort. (B) Violin plot of top 10 antigen‐related genes in the *CXCL11*‐positive and *CXCL11*‐negative groups (Wilcoxon signed rank test, ***p < .001). (C) Correlation between *CXCL11* expression signature and neoantigen load in the TCGA‐OSC cohort

The results from the TIMER and GSEA analysis showed that there were significant differences between the *CXCL11*‐positive and the *CXCL11*‐negative groups in antigen processing and presentation pathway, hinting that the expression of antigen‐related genes might be associated with *CXCL11* expression. To prove this assumption, we explored the correlation of antigen‐related genes with *CXCL11* expression by using the Pearson correlation coefficient. We found that the expression of MHC class I/II (I: HLA‐A, HLA‐B, and HLA‐C; II: HLA‐DP, HLA‐DM, HLA‐DOA, HLA‐DOB, HLA‐DQ, and HLA‐DR) and antigen binding (B2M, TAP1/2, and so on) molecules were highly correlated with the *CXCL11* expression signature (Figure 6A). There were significant differences in the expression of HLA‐A, HLA‐B, and other key antigen presenting molecules between the two subgroups (Wilcoxon signed rank test, *p* < .001, Figure 6B). The results were confirmed in the GEO validation cohort (Figure S4). As antigen processing and presentation pathway plays a crucial role in immune recognition of predicted (neo‐) antigen produced by cancer cells, we further investigated the relationship between neoantigen load and the *CXCL11* expression signature by Pearson correlation coefficient. Correspondingly, predicted neoantigen load was highly correlated with *CXCL11* expression (Figure 6C).

### 3.6 *CXCL11* expression associated with ICB‐related genes

**FIGURE 7:**
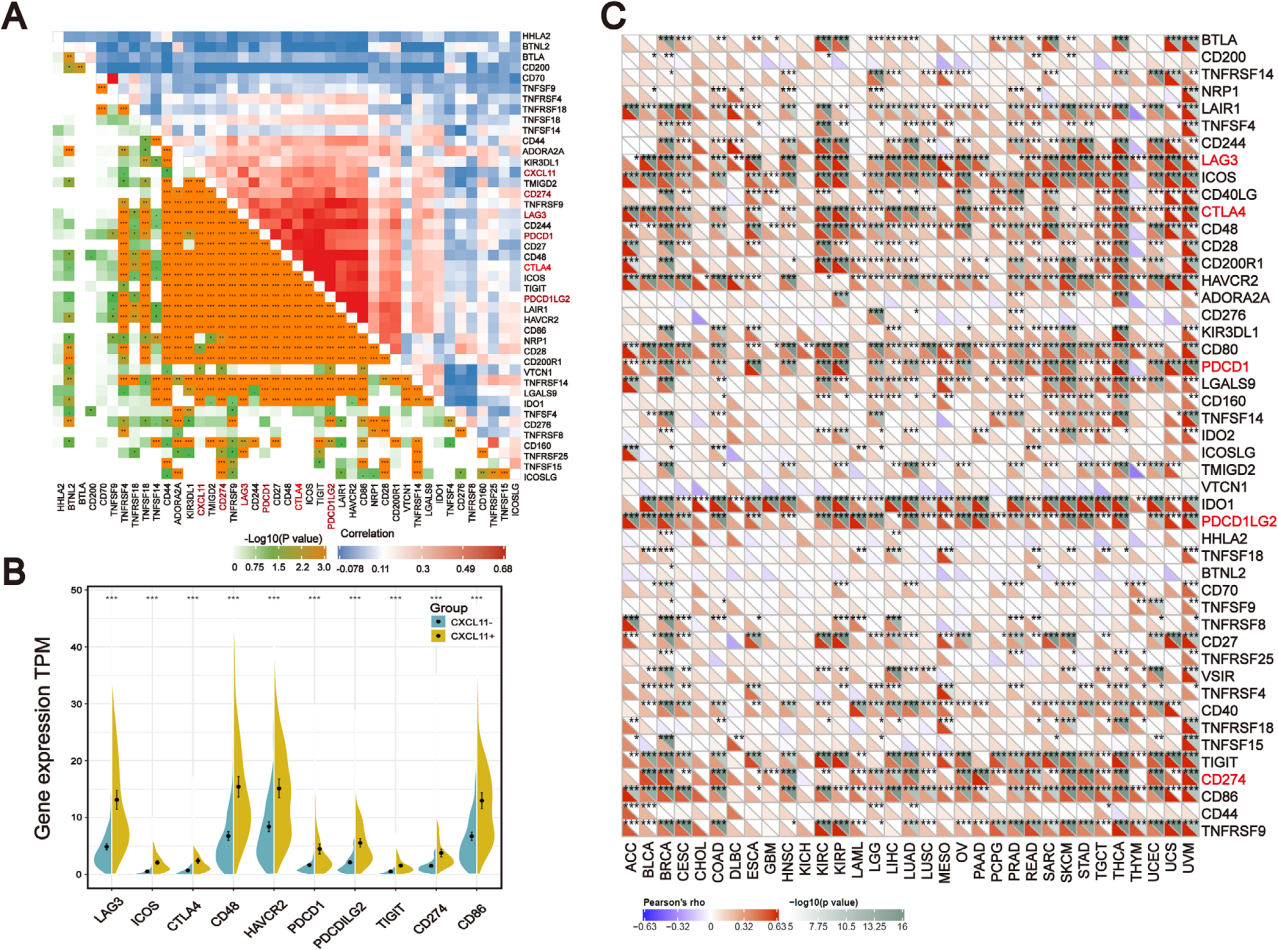
Correlation among the expression of *CXCL11* and ICB‐related genes. (A) Correlation between the *CXCL11* expression signature and ICB‐related genes in the TCGA‐OSC cohort. (B) Violin plot of top 10 ICB‐related genes in the *CXCL11*‐positive and *CXCL11*‐negative groups (Wilcoxon signed rank test, ***p < .001). (C) Correlation between *CXCL11* expression signature and ICB‐related genes in the TCGA‐pan cancer cohorts

In recent years, ICB therapy, represented by anti‐PD‐1/L1, has played an increasingly important role in antitumor treatment. The characteristics of TIME and immune checkpoint genes in tumor cells have a profound impact on ICB therapy. Therefore, we collected more than 40 common ICB‐related gene signatures and analyzed the relationship between *CXCL11* expression and ICB‐related genes.^35^ As displayed by heatmap, *CXCL11* expression was significantly correlated with the expression of multiple ICB‐related genes (Figure 7A). Ten of the most relevant ICB‐related genes were: *LAG3*, I*COS, CTLA4, CD48, HAVCR2, PDCD1* (PD‐1)*, PDCDILG2* (PD‐L2)*, TIGIT, CD274* (PD‐L1), and *CD86*, and their expression levels were enriched in the *CXCL11*‐positive group (Wilcoxon signed rank test, *p* < .001, Figure 7B). Generally, the key regulatory factors involved in immunity perform similar functions in different tissues.^36^ We thus explored *CXCL11* expression and ICB‐related gene signatures across cancer types. We found that the coexpression of *CXCL11* and ICB‐related genes was not only present in ovarian cancer, but also in 32 other cancer types (Figure 7C).

### 3.7 *CXCL11* expression could be used as a potential biomarker for ICB therapy

**FIGURE 8:**
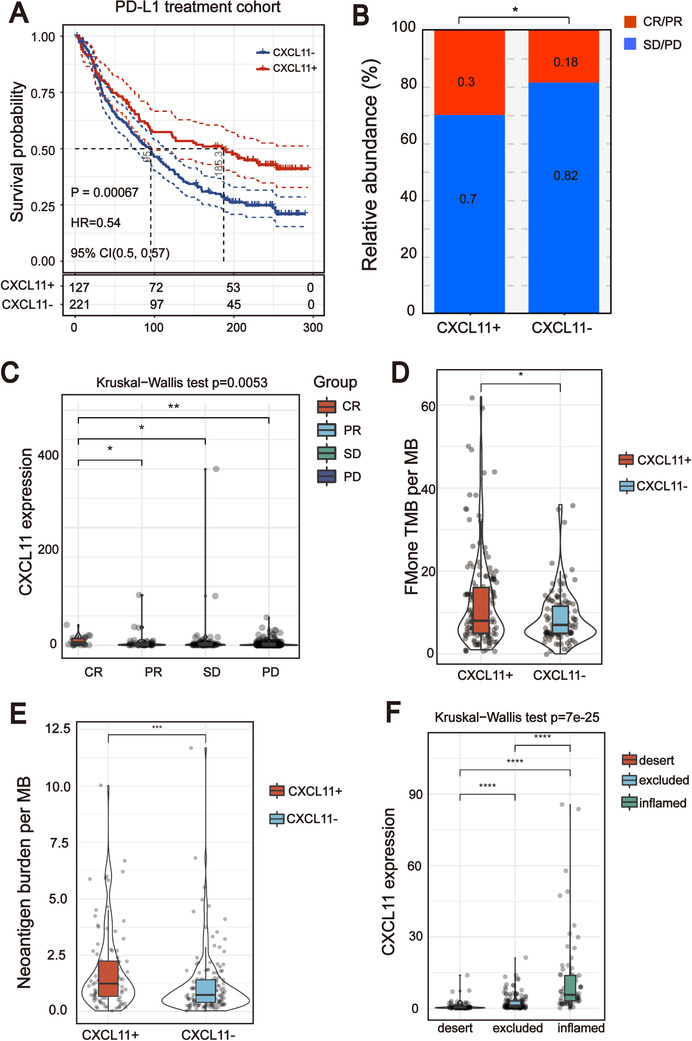
*CXCL11* expression could be used as a potential biomarker for ICB therapy. (A) Curve for overall survival is shown for high and low *CXCL11* expression in the PD‐L1 treatment cohort. (B and C) Proportion of immune response to anti‐PD‐L1 treatment in high versus low *CXCL11* expression subgroups. CR, complete response; PD, progressive disease; PR, partial response; SD, stable disease. (D) TMB and neoantigen load (E) in the immunotherapy cohort were compared among distinct *CXCL11* expression signature subgroups. (F) *CXCL11* expression signature in different immune phenotype subgroups. The tumor immunophenotype was defined according to immunohistochemistry results of the CD8 antibody (Wilcoxon signed rank test, ****p < .0001)

All of the above results indicate that *CXCL11* expression is closely related to the biomarkers for ICB therapy. Therefore, we collected the transcriptome profile and clinical information from an immunotherapy cohort (Imvigor210) of urothelial cancer treated with atezolizumab, so as to explore the relationship between *CXCL11* expression and immune response.^14^ In this cohort, tumor patients with high *CXCL11* expression exhibited markedly improved clinical benefits and significantly prolonged survival (Figure 8A). Significant therapeutic advantages and immune responses to PD‐L1 blockades were observed in samples with high expression of *CXCL11* compared to those with low expression (Fisher extract test, *p* = .0002, Figure 8B; Kruskal–Wallis *H* test, *p* < .001, Figure 8C). Further analysis revealed that tumor infiltrating immune phenotype and neoantigen load were significantly elevated in tumors with high expression of *CXCL11*, which was closely linked to immunotherapeutic efficacy (Figures 8D,E). Besides, the association between the expression of *CXCL11* and immunotherapy survival remained statistically significant after taking into account gender, smoking, ECOG score, immunophenotype, and PD‐1/PD‐L1 status (Figure S5).

### 3.8 Olaparib‐treated ovarian cancer cells upregulate *CXCL11* expression in vivo and in vitro

**FIGURE 9:**
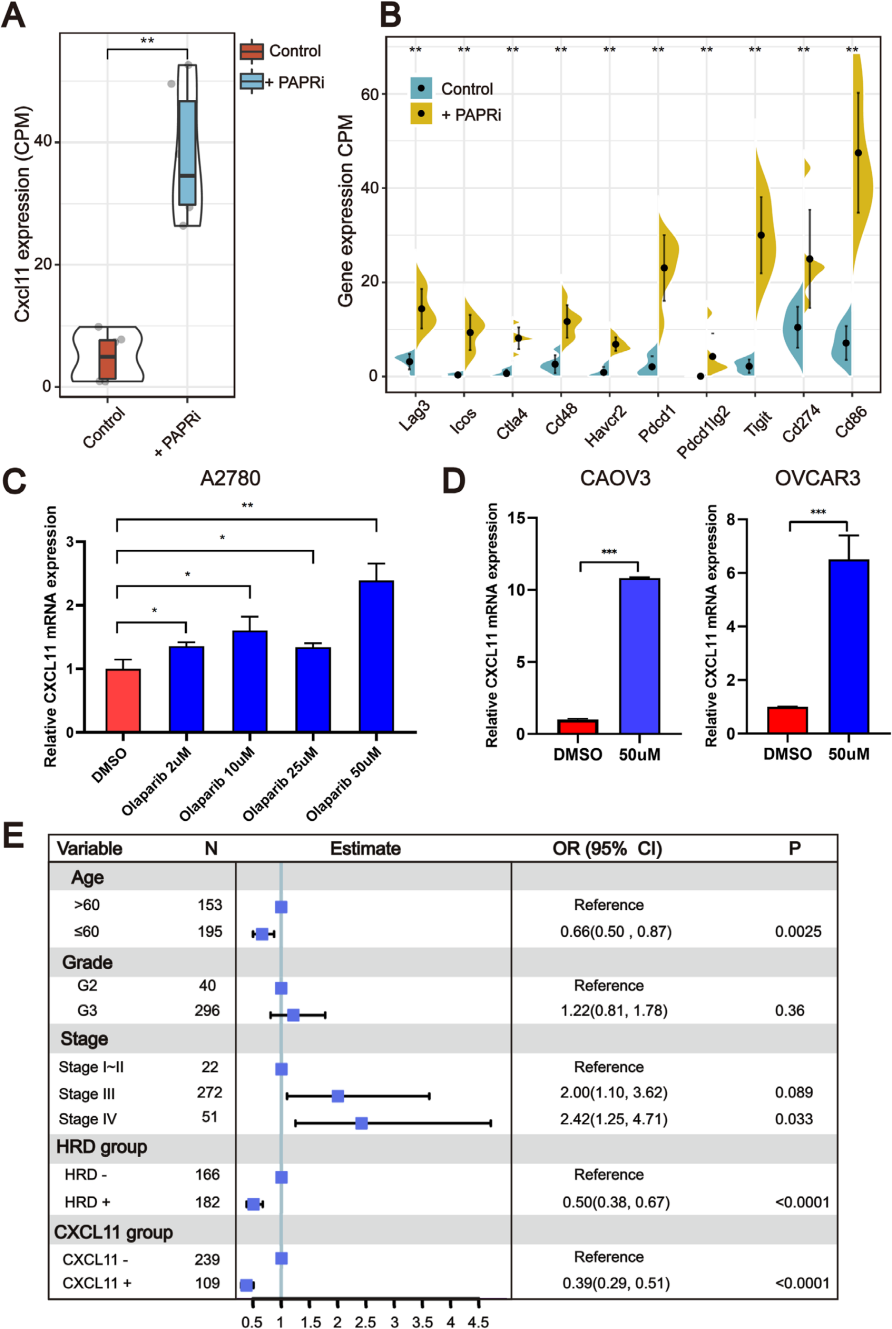
Olaparib elicits the expression of *CXCL11* in vivo and in vitro. (A and B) Olaparib elicits the expression of *CXCL11* and ICB‐related genes in vivo (Wilcoxon signed rank test, **p < .01). (C and D) qPCR evaluation of *CXCL11* expression in different cell lines. Olaparib elicits the expression of *CXCL11* in multiple ovarian cancer cell lines (Student's *t*‐test, *p < .05, **p < .01). (E) Forest plot representation of the multivariate Cox regression model delineated the association between the *CXCL11* expression signature and survival in the TCGA‐OSC cohort

It has been reported that PARP inhibitor (PARPi) treatment markedly induced DSBs. To confirm that upregulated *CXCL11* expression could be derived from HRD tumor cells, we first re‐analyzed the RNA seq data of high‐grade serous ovarian cancer tumor tissues harvested from tumor‐bearing mice after 18 days of treatment with olaparib or vehicle (GSE120500).^37^ Boxplots showed markedly upregulated expression of *CXCL11* in tumors treated with olaparib compared with vehicle control (Figure 9A).

We next compared the expression levels of ICB‐related genes with the highest correlation with *CXCL11* between the olaparib treatment group and the control group. As shown in Figure 9B, the expression of these genes in the olaparib treatment group was significantly higher than that in the vehicle control group (Wilcoxon signed rank test, *p* < .01). To further validate that genomic instability ovarian cancer cells activate the *CXCL11* expression signature, we conducted in vitro experiments. As measured by RT‐qPCR (Figure 9C,D), olaparib treatment caused significant upregulation of *CXCL11* mRNA expression in multiple ovarian cancer cell lines. Together, these data indicate that cancer cells with DSBs could upregulate the expression of *CXCL11* in vivo and in vitro.

## 4 DISCUSSION

Over the years, many efforts have been made to investigate the initiation, development, and treatment of ovarian cancer.^38^ Postoperative histopathological characteristics of patients such as tumor size, stage and grade, and residual lesions are still used as the most important prognostic factors for ovarian cancer. However, the 5‐year relative survival rate of ovarian cancer patients is still unsatisfactory. HRD has been reported to be not only a ubiquitous feature of breast cancer but is also one of the most influential factors for ovarian cancer prognosis. However, HRD is present in less than 50% of serous ovarian tumors, so new biomarkers need to be developed for molecular typing of ovarian cancer patients. In this study, we deeply analyzed the molecular characteristics of OSC patients with different HRD scores and identified biomarkers that could be complementary to the HRD score, our contributions are as follows:

(1) A comprehensive analysis of the genomic landscape of non‐HRD group showed a high proportion of actionable gene mutations. (2) We found that *CXCL11* expression, a downstream target of the cGAS‐STING pathway, was positively associated with HRD and displayed a strong ability to predict the prognosis of OSC patients. (3) We introduced *CXCL11* as a potential reliable biomarker for the efficacy of ICB therapy, and the predictive effect of *CXCL11* was even better than that of PD‐1/PD‐L1.

The basket study design is noteworthy because it allows for the possibility that different tumor types with the same molecular biomarker might differ in their sensitivity to therapy targeted at that biomarker.^39^ Potentially actionable mutations were seen in 13% of non‐HRD patients: (a) loss‐of‐function mutations in *NF1* were found in 8% of the patients; (b) loss‐of‐function mutations in *CDK12* were found in 5% of the patients. The *NF1* gene encoding neurofibromin works as a negative regulator of RAS activity. Patients with *NF1* gene loss‐of‐function mutations are more likely to develop RAS hyperactivity and tumorigenesis.^40^, ^41^ The availability of small molecule compounds (such as selumetinib and imatinib) that target RAS signaling implied in the pathogenesis of plexiform neurofibromas has led to multiple clinical trials, and FDA has approved Koselugo (selumetinib) for the treatment of pediatric patients with *NF1* mutations.^42^ CDK12 (cyclin‐dependent kinase 12) is a kinase involved in regulation of the cell cycle and regulation of transcriptional elongation of many DNA‐damage‐response genes. Loss of the CDK12/cyclin K complex renders triple‐negative breast cancer and HEK293 cells sensitive to various DNA‐damaging agents, including camptothecin, etoposide, and mitomycin C.^43^ Comprehensive genomic analysis of non‐HRD ovarian cancer has broadened our knowledge of the molecular events relevant to patients who cannot receive olaparib plus bevacizumab treatment, and provides a direction for targeted therapy of these patients.

Through the whole transcriptome analysis of the patients with HRD tumors, we identified that *CXCL11* expression could be a reliable prognostic risk gene in the TCGA‐OSC cohort and its efficacy was proved in the GEO‐OSC cohorts. *CXCL11* is a small cytokine belonging to the CXC chemokine family.^44^ As a downstream target of cGAS‐STING, *CXCL11* is a critical chemokine that binds CXCR3 on T cells, regulating differentiation of naive T cells and leading migration of immune cells to their focal sites.^45^, ^46^ In the recent issue of *Cancer Cell*, Lu and Guan demonstrated that activation of the cGAS‐STING pathway in tumor tissues was significantly and positively correlated with the prognosis of patients bearing dMMR tumors but not that of patients with pMMR (proficient MMR) tumors. In addition to dMMR, HRD also induces genomic instability and serves as an effective therapeutic biomarker for breast cancer and ovarian cancer.^47^, ^48^ As a complement to Guan and Lu's work, our results further demonstrated that the correlation between genomic instability and activated cGAS‐STING signaling in dMMR tumors may be extended to HRD tumors.

Emerging evidence has shown the importance of CXC chemokines in tumor immunotherapy.^49^, ^50^ Our results showed that *CXCL11* expression was positively associated with TIME, including neoantigen load and infiltrating immune cells, and could be used as a potential biomarker for ICB therapy.

Under this situation, the determination of whether upregulated *CXCL11* unavoidably results from the cGAS‐STING activation needs further experimental testing. As a downstream target of the cCAS‐STING pathway, upregulated *CXCL11* showed superior predictive power compared with the HRD score. Importantly, the clinical examination of *CXCL11* in tumor tissues or serum is more feasible than applying the steps necessary for calculating the HRD score, and the prediction accuracy of upregulated *CXCL11* is even better than the HRD score itself. We introduced, for the first time, a prospective biomarker associated with HRD tumors, which merits further investigation in multiple cohorts.

## 5 CONCLUSIONS

To summarize, this work provided a new perspective on the molecular characteristics of the genomic and transcriptome of patients with OSC. Our results were the first to find that the non‐HRD patients had the opportunity for targeted therapy, laying the foundation for molecular typing of OSC. Furthermore, this work identified the *CXCL11* expression signature that could not only predict OSC patients’ survival outcomes but also work as a potential reliable biomarker for the efficacy of ICB therapy. Our study showed high clinical application value and provided new clues for enrolling OSC patients in precision medicine. Through further prospective validation and mechanism research, biomarkers derived from this work may become important molecules for molecular typing of OSC.

## CONFLICT OF INTEREST

The authors declare that there is no conflict of interest.

## AUTHOR CONTRIBUTIONS

Designing research studies: Zhiwen Shi and Keqin Hua. Conducting experiments: Junjun Qiu, Qingguo Zhao, and Bin Lv. Analyzing data: Zhiwen Shi and Hongyan Wang. Preparing the manuscript: Zhiwen Shi and Junjun Qiu. Grammar check: Xinyu Qu and Xiao Han. Supervision: Hongyan Wang, Junjun Qiu, and Keqin Hua. Funding acquisition: Keqin Hua and Junjun Qiu. The authors read and approved the final manuscript.

## Supporting information

Supporting information
**Supporting Figure S1** Molecular characteristics of patients in the HRD and non‐HRD groups. (A) GSEA identified that DNA replication, homologous recombination, and mismatch repair signaling pathways were upregulated in the HRD group compared to non‐HRD group. (B) Violin plot of MSI sensor score in the HRD and non‐HRD groups. (C) Venn diagram showing the shared genes between the HRD and non‐HRD groups

Supporting information
**Supporting Figure S2** Mutations in the homologous recombination pathway of the HRD and non‐HRD groups. (A and B) Genetic profile of the HRD and non‐HRD patients in the homologous recombination pathway. (C) Copy number variation in homologous recombination pathway of patients in the HRD and non‐HRD groups

Supporting information
**Supporting Figure S3** *CXCL11* expression signature was associated with OSC patients’ survival in the GEO validation cohorts. (A and B) Kaplan–Meier estimates of OS of patients with the *CXCL11*‐positive or *CXCL11*‐negative tumors in the GEO validation cohorts (log‐rank test). (C) Correlation between the *CXCL11* expression signature and immune cell subpopulations in the TCGA pan‐cancer cohorts

Supporting information
**Supporting Figure S4** Correlation between the expression of *CXCL11* and MHC molecules in the GEO validation cohort. (A) Correlation between the *CXCL11* expression signature and MHC molecules in the GSE140082 cohort. (B) Violin plot of HLA molecules associated with antigen presentation in the *CXCL11*‐positive and *CXCL11*‐negative groups (Wilcoxon signed rank test, ***p < .001)

Supporting information
**Supporting Figure S5** Multivariate Cox regression analysis of the *CXCL11* expression signature with gender, smoking, ECOG score, and immunophenotype were taken into account

Supporting information

Supporting information
