# Supplementary figures and images for: Identification of biomarkers complementary to homologous recombination deficiency for improving the clinical outcome of ovarian serous cystadenocarcinoma
Source: Clin Transl Med. 2021 May 18;11(5):e399. doi: 10.1002/ctm2.399 (PMC8131501; doi:10.1002/ctm2.399)

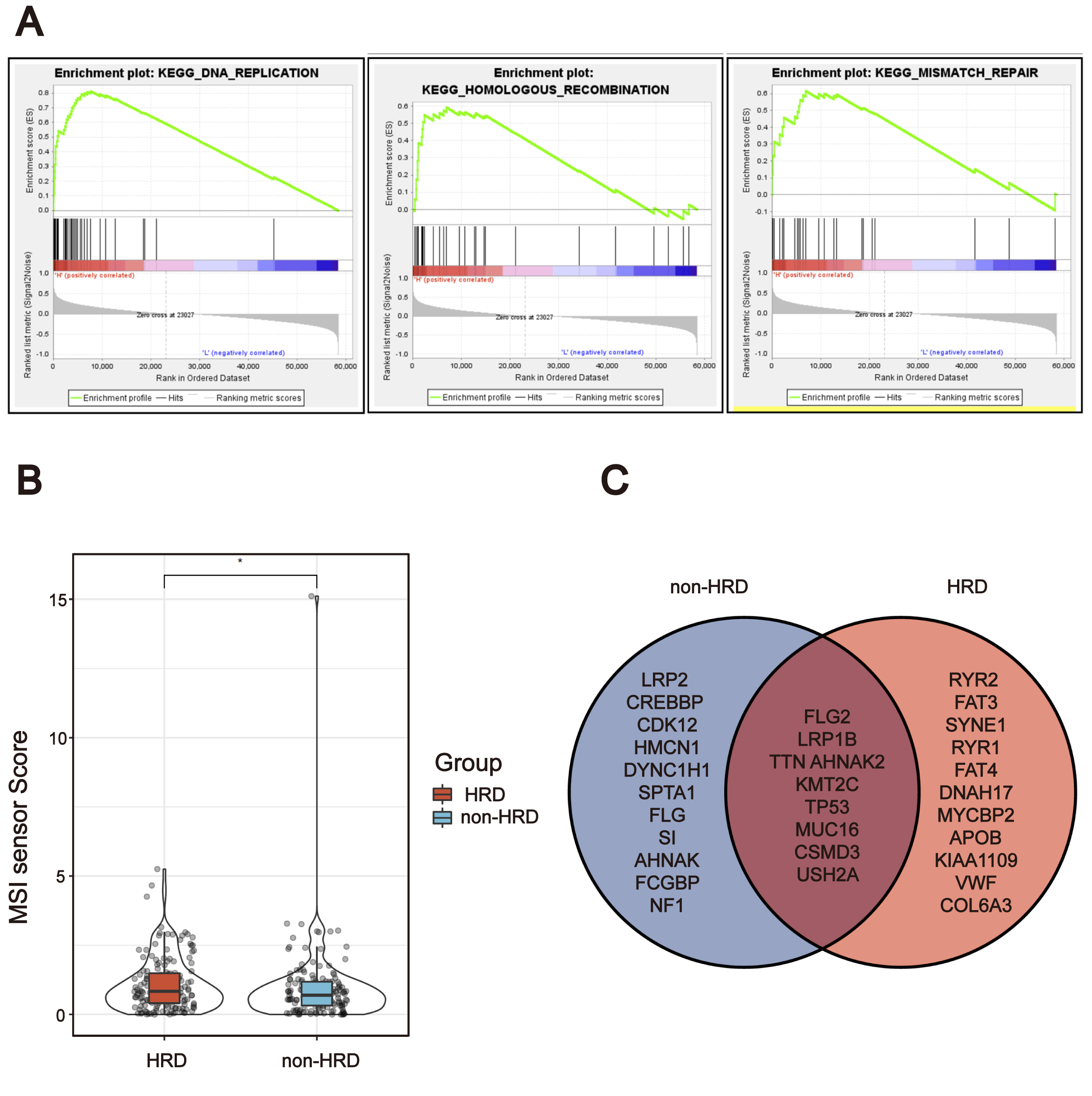

Supplement: Supplementary file 1 — Supporting information Supporting Figure S1 Molecular characteristics of patients in the HRD and non‐HRD groups. (A) GSEA identified that DNA replication, homologous recombination, and mismatch repair signaling pathways were upregulated in the HRD group compared to non‐HRD group. (B) Violin plot of MSI sensor score in the HRD and non‐HRD groups. (C) Venn diagram showing the shared genes between the HRD and non‐HRD groups [file CTM2-11-e399-s006.tif]

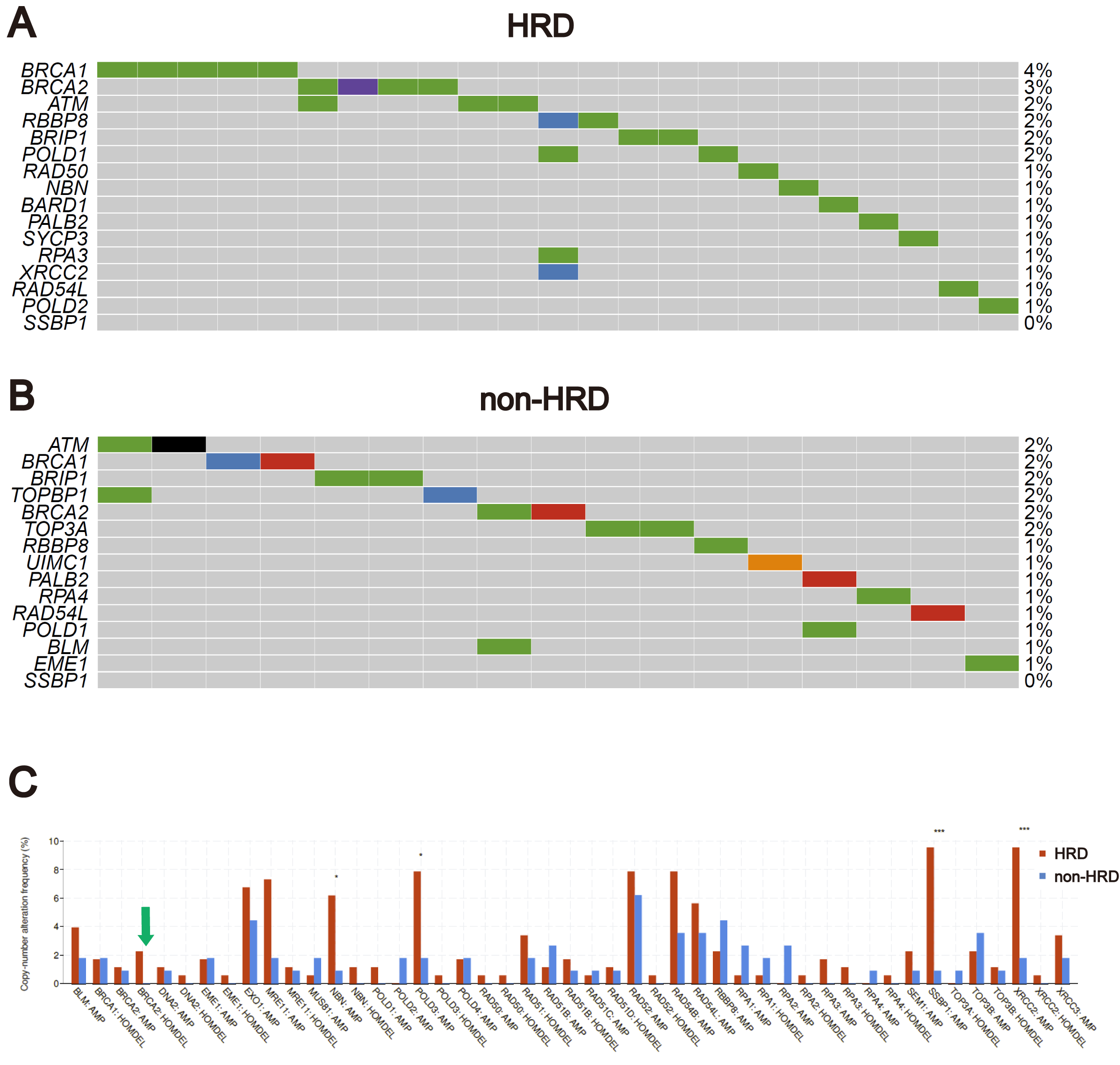

Supplement: Supplementary file 2 — Supporting information Supporting Figure S2 Mutations in the homologous recombination pathway of the HRD and non‐HRD groups. (A and B) Genetic profile of the HRD and non‐HRD patients in the homologous recombination pathway. (C) Copy number variation in homologous recombination pathway of patients in the HRD and non‐HRD groups [file CTM2-11-e399-s001.tif]

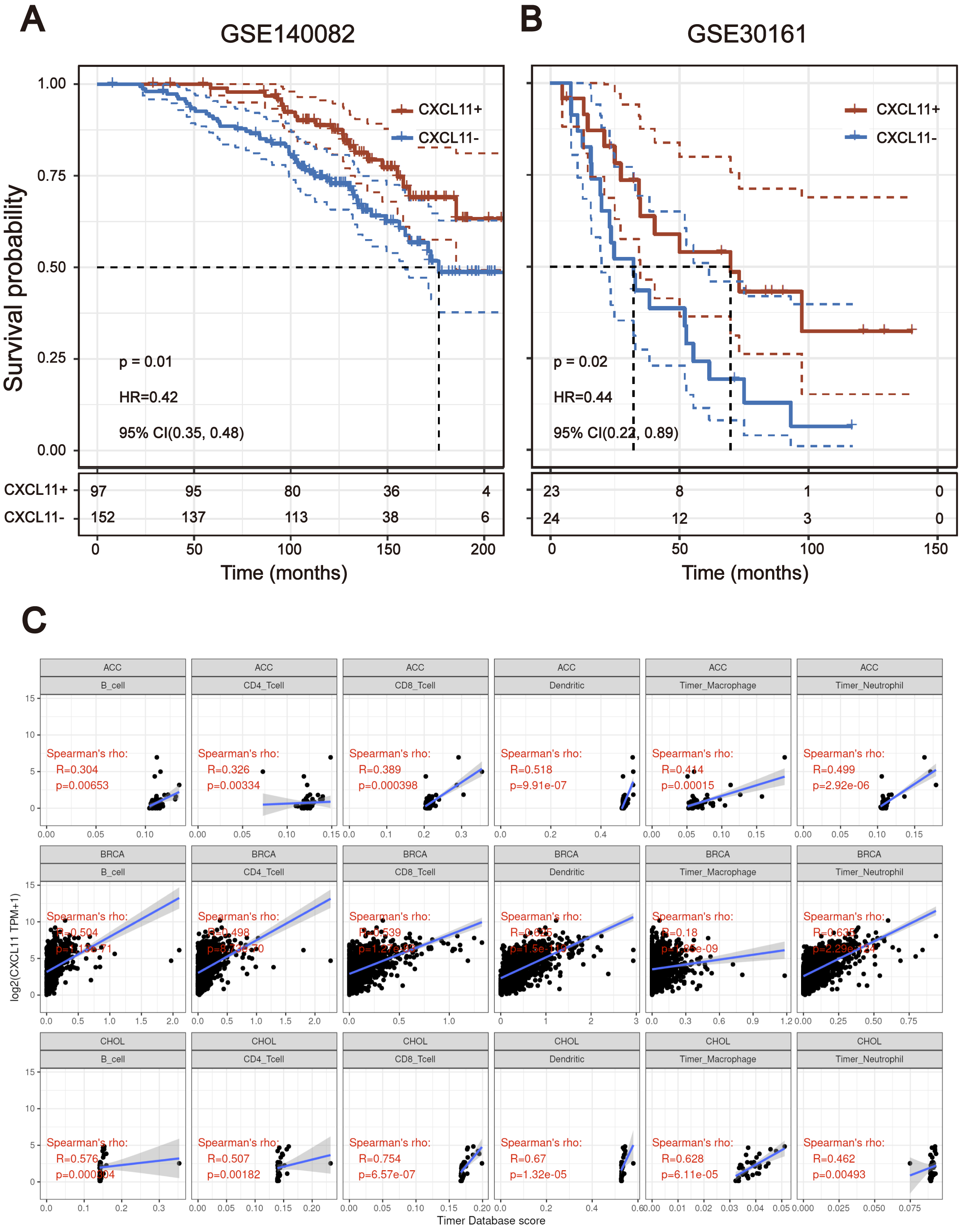

Supplement: Supplementary file 3 — Supporting information Supporting Figure S3 CXCL11 expression signature was associated with OSC patients’ survival in the GEO validation cohorts. (A and B) Kaplan–Meier estimates of OS of patients with the CXCL11‐positive or CXCL11‐negative tumors in the GEO validation cohorts (log‐rank test). (C) Correlation between the CXCL11 expression signature and immune cell subpopulations in the TCGA pan‐cancer cohorts [file CTM2-11-e399-s004.tif]

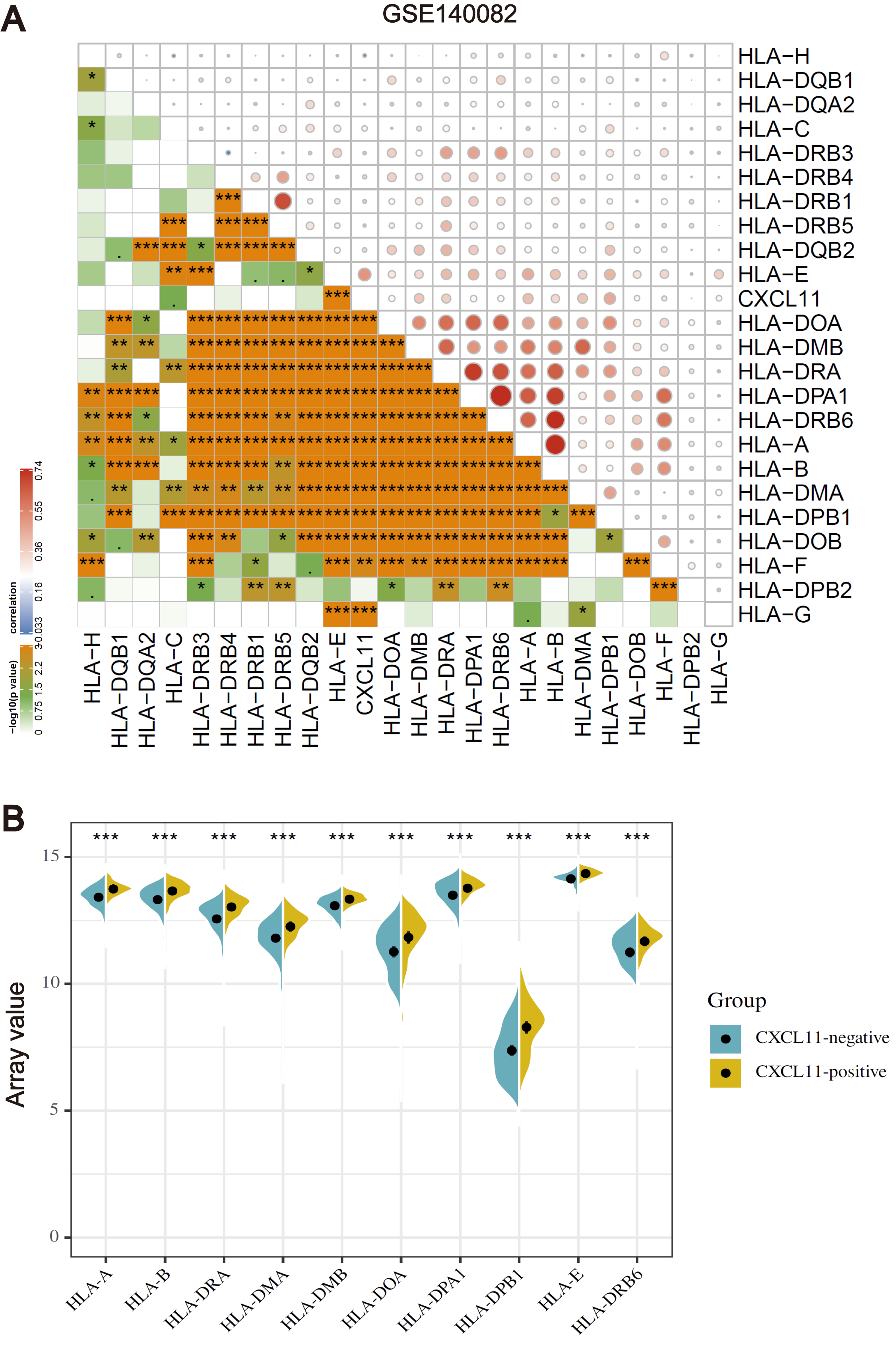

Supplement: Supplementary file 4 — Supporting information Supporting Figure S4 Correlation between the expression of CXCL11 and MHC molecules in the GEO validation cohort. (A) Correlation between the CXCL11 expression signature and MHC molecules in the GSE140082 cohort. (B) Violin plot of HLA molecules associated with antigen presentation in the CXCL11‐positive and CXCL11‐negative groups (Wilcoxon signed rank test, ***p < .001) [file CTM2-11-e399-s005.tif]

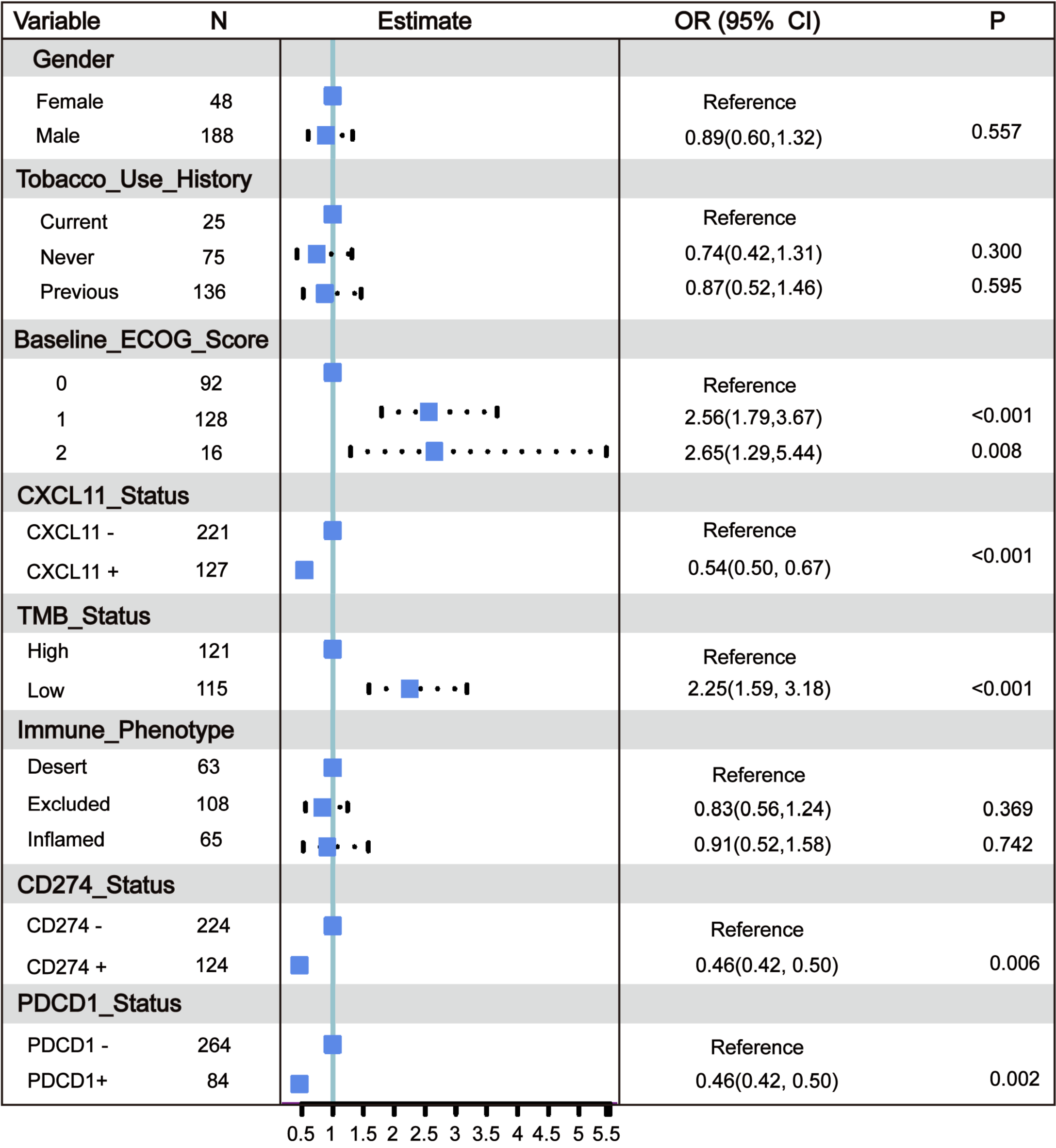

Supplement: Supplementary file 5 — Supporting information Supporting Figure S5 Multivariate Cox regression analysis of the CXCL11 expression signature with gender, smoking, ECOG score, and immunophenotype were taken into account [file CTM2-11-e399-s002.tif]
